# Lifetime Psychotic Symptoms, Subthreshold Depression and Cognitive Impairment as Barriers to Functional Recovery in Patients with Bipolar Disorder

**DOI:** 10.3390/jcm8071046

**Published:** 2019-07-18

**Authors:** Caterina Mar Bonnín, Esther Jiménez, Brisa Solé, Carla Torrent, Joaquim Radua, María Reinares, Iria Grande, Victoria Ruíz, Jose Sánchez-Moreno, Anabel Martínez-Arán, Eduard Vieta

**Affiliations:** 1 Barcelona Bipolar Disorders and Depressive Unit, Hospital Clinic, Institute of Neurosciences, University of Barcelona, IDIBAPS, CIBERSAM, Barcelona, 08036 Catalonia, Spain; 2 Department of Psychosis Studies, Institute of Psychiatry, Psychology and Neuroscience, King’s College London, London SE5 8AF, UK; 3 Department of Clinical Neuroscience, Centre for Psychiatry Research, Karolinska Institutet, 113 30 Stockholm, Sweden

**Keywords:** bipolar disorder, psychosocial functioning, psychotic symptoms, depressive symptoms

## Abstract

(1) Background: bipolar disorder (BD) is a chronic disease that often leads to functional impairment. The objective of this study is to elucidate which variables are associated with better functional outcomes in a sample of euthymic patients with BD. (2) Methods: patients were recruited at the Hospital Clinic of Barcelona and they underwent a clinical interview, a functional assessment, and a comprehensive neuropsychological evaluation. After that, patients were divided into two groups according to the Functioning Assessment Short Test total score: functionally remitted vs. functionally impaired. Following this, a multivariate logistic regression was run in order to identify clinical, demographic and cognitive factors associated with functional remission. (3) Results: a total of 420 euthymic patients with BD were assessed for this study, distributed as follows: functionally remitted (*n* = 221) and functionally impaired (*n* = 199). Finally, the multivariate logistic regression revealed that only five variables significantly contributed to the model, including: lifetime history of psychotic symptoms (the variable that contributed the most to the model), followed by the Hamilton Depression total score, and cognitive performance (executive functions and verbal memory). (4) Conclusions: treatments to ensure a good functional outcome in BD should specially prevent psychosis, target subthreshold depressive symptoms and enhance cognition, more specifically executive functions and verbal memory.

## 1. Introduction

Bipolar disorder (BD) is a lifelong illness that often results in functional impairment [1,2]. Clinical outcomes in BD have been traditionally defined in the context of response, partial response and nonresponse. However, over the last years functional recovery has become a major issue and one of the most desired outcomes in clinical settings [3]. It is expected that after the resolution of an acute episode patients regain their functioning in all areas of their lives, including household tasks, performance at school/work, and interpersonal relationships. Nonetheless, the reality is quite different from this and researchers have been reporting for many years that a substantial proportion of patients with BD suffer from functional impairment. It has been found that between 30–60% of patients do not reach functional recovery during euthymia [4,5,6]. In this regard, a recent study has investigated the factors associated with poor functional outcome in a sample of patients with marked functional impairment. The authors found that male gender, older age, lower premorbid intelligence quotient (IQ), subclinical depressive symptoms, a higher number of manic episodes and lower performance in some neuropsychology tests (working memory, verbal fluency, verbal memory and processing speed) were associated with poorer functional outcomes [7]. Similarly, another study reported that patients with low psychosocial functioning exhibited an impaired cognitive profile characterized by deficits in processing speed and some variables of executive functions, together with high levels of residual symptomatology and higher rates of unemployment [5]. Identifying the variables associated with functional impairment might be crucial to prevent the functional decline typically seen in some patients with BD and improve their quality of life. To achieve this objective, an adequate tool to assess functioning is also essential as a way to capture the functional outcome in BD. In 2007, the Bipolar Disorders and Depression Unit at the Hospital Clinic (Barcelona) developed a scale called The Functioning Assessment Short Test (FAST). The FAST tackles the most frequent difficulties reported by patients with BD covering up to six different domains including autonomy, occupational and cognitive functioning, interpersonal relationships, financial issues and leisure time. It also provides a total global score of functional impairment [8]. More recently, the same group of research has provided a classification of severity based on the total global score of the FAST which were estimated taking into account the Global Assessment of Functioning (GAF) scores as a reference, and as a result, four categories were identified: none, mild, moderate and severe impairment [6]. Indeed, this classification may be useful not only to measure clinically meaningful changes in functioning after a given treatment, but also to study the variables associated to each of the above-mentioned categories. So far, no studies have focused on the variables associated with functional recovery using the classification of severity provided by the FAST scale and most studies to date are limited as regards to sample size. The aim of the present report is to study demographic, clinical and neurocognitive variables associated with functional recovery and remission in a large sample (more than 400 patients) of euthymic patients suffering from BD. 

## 2. Experimental Section

### 2.1. Participants

Participants with BD were recruited at the Bipolar Disorders and Depression Unit from the Hospital Clinic of Barcelona. This hospital-based program provides integrated care for difficult-to-treat patients with BD from across Catalonia, as well as care to patients with BD from a specific catchment area in Barcelona [9,10]. The patients fulfilled the following inclusion criteria: (a) diagnosed with BD according to the Diagnostic and Statistical Manual of Mental Disorders- Text Revision (DSM-IV-TR) criteria; (b) assessed during euthymia, defined as the Young Mania Rating Scale (YMRS) [11,12] score < 6 and Hamilton Depression Rating Scale-17 (HAM-D) [13,14] score < 8; (c) aged between 18 and 70 years old. Exclusion criteria were: (a) current diagnosis of substance abuse or dependence; (b) history of mental retardation or any clinical condition that could interfere in the interview; (c) estimated IQ lower than 85.

This study was conducted in accordance with the ethical principles of the Declaration of Helsinki and Good Clinical Practice and the study protocol was approved by the ethics committee of the Hospital Clinic. All participants received extensive information on the study and provided written informed consent prior to enrolment.

### 2.2. Clinical and Functional Assessments

After providing written informed consent, all participants went through a structured clinical interview of the Program’s protocol based on the Structured Clinical Interview for DSM-IV-TR [15]. Variables such as age, gender, diagnosis, number and type of episodes, chronicity (illness duration in years), number of hospitalizations, history of psychosis, history of rapid cycling and family affective psychiatric history were collected. All the information provided by the patients was complemented with information from the clinical records.

After collecting these data, patients were assessed with several clinical scales: first, the HAM-D and the YMRS were administered to ensure that patients met criteria for euthymia at the time of the assessment. Psychosocial functioning was assessed by means of the FAST [8]. As explained in the introduction, the FAST scale is an interviewer-administered instrument developed to assess the main difficulties in daily life of patients with BD. The global score is the addition of the score from the 24 items comprising the scale. The total score can range from 0 to 72 with higher scores indicating greater disability. A recent study has provided different cut-offs scores to classify patients in none, mild, moderate and severe impairment depending on the total score of the FAST scale [6]. More specifically, in that study, the cut-offs for each category of severity were stablished as follows: patients scoring from 0 to 11 in the global score were classified as non-impaired; patients scoring between 12 and 20 were mildly impaired; patients scoring from 21 to 40 exhibited moderate impairment; finally, patients scoring above 40 were classified as severely impaired. For the present study, patients were divided into two groups following this classification: (1) the functionally remitted group comprised the first two categories, that is the non and mild impairment; hence, this category included patients who scored between 0 and 20 in the FAST global score; (2) the functionally impaired group comprised patients from the moderate and severe category, that is, patients scoring between 21 and 72 in the FAST global score. 

### 2.3. Neuropsychological Assessment

Participants were assessed using a comprehensive neuropsychological battery. This assessment involved different tests described as follows: estimated IQ was evaluated with the Wechsler Adult Intelligence Scale (WAIS-III) [16], vocabulary subtest. The processing speed index consisted of two subtests of the WAIS-III [16]: the digit-symbol coding and symbol search. The working memory index comprised the arithmetic, digits, and letter-number sequencing subtests of the WAIS-III [16]. Verbal memory was assessed with the California Verbal Learning Test (CVLT) [17]. Executive functions were tested by several tests assessing set shifting, planning, verbal fluencies, and response inhibition, namely the computerized version of the Wisconsin Card Sorting Test [18], the Stroop Color-Word Interference Test (SCWT) [19], the Trail Making Test-part B (TMT-B) [20], phonemic fluency (F-A-S) and categorical fluency (animal naming), both components of the Controlled Oral Word Association Test (COWAT) [21]. Visual learning and memory were assessed by means of the Rey–Osterrieth Complex Figure (ROCF) [22]. Finally, attention was examined with the Trail Making Test-part A (TMT-A) [20]. 

### 2.4. Statistical Analyses

The first step of these analyses was to calculate the total global score of the FAST for each participant in the study. After that, they were classified as functionally remitted (whenever the score was twenty or below) or as functionally impaired (when the participant scored twenty-one or above). Then, descriptive analyses of the two groups were performed using Chi-square tests for categorical variables and Student *t* tests for continuous variables. These analyses included demographic, clinical and neuropsychological variables. The raw scores from the neurocognitive variables were converted into z-scores for a better comparison in the different tests applied. Since there is no control group, z scores were performed taking into account the mean and the standard deviation of the whole sample of patients with BD. 

After the descriptive analyses, the multivariate logistic regression model was performed. Logistic regression was used to estimate the effects of the risk factors associated with functional impairment. Variables were selected for inclusion in logistic regression when significance at *p* < 0.05 in the univariate analysis was met (including clinical, demographic or neuropsychological variables). Data were analyzed using SPSS v.23 for Windows (Chicago, IL, USA). All analyses were two-tailed with alpha set at *p* < 0.05. 

## 3. Results

### 3.1. Demographic and Clinical Features

A total of 420 participants were recruited for this study. According to the severity criteria they were classified in the functionally remitted group (*n* = 221) vs. the functionally impaired group (*n* = 199). As shown in Table 1, both patient groups differed in terms of age, gender, HAM-D total score, number of previous depressive episodes, years of illness, and number of hospitalizations. In this regard, patients in the functionally impaired group presented higher scores in HAM-D total score, had suffered from more depressive episodes and previous hospitalizations; the impaired group also presented more years of illness duration (chronicity) Regarding demographic variables, patients in the impaired group were older and more prevalent in male gender. For further details, see Table 1. 

### 3.2. Neuropsychological Performance

**Figure 1:**
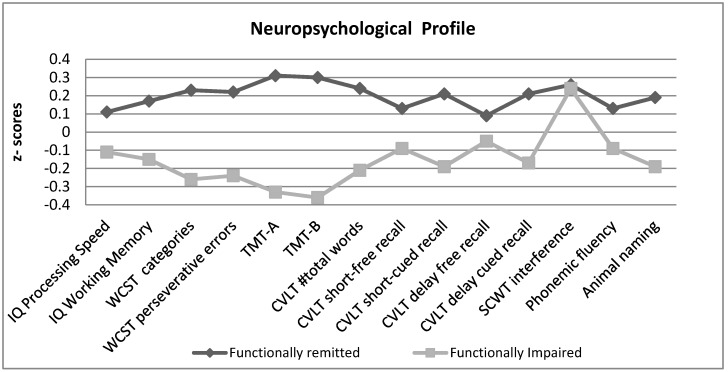
Neuropsychological profile in the two patient groups (normalized z-scores).

In general, the neuropsychological performance of the patients in the functionally impaired group was significantly lower compared to those with no functional impairment, except for two variables: the CVLT delay free recall (*t* = 1.4, *p* = 0.15) and SCWT interference (*t* = 0.47; *p* = 0.63), where patients in both groups performed similarly. Table 2 shows the scores in all the assessed variables, and Figure 1 compares the neuropsychological performance between the two groups using standardized variables (z-scores).

### 3.3. Identifying Factors Associated with Functional Recovery

A logistic regression analysis was performed to assess the role of the variables on the likelihood that patients presented functional impairment, as measured with FAST categorization. The variables included the model comprised of all those that were found to be significant when comparing both groups (impaired vs. non-impaired) in demographic and clinical variables (see Table 1) and in the neuropsychological performance (see Table 2). Besides these variables, the authors included other relevant variables in the analysis that have been found to be related with psychosocial impairment according to the literature; these relevant variables included: diagnosis subtype (bipolar type I vs. type II); presence of lifetime psychosis, estimated premorbid IQ and number of manic episodes. 

After running the logistic regression with all these variables, it was found that the final model included only five significant variables explaining between 35.6% (Cox and Snell R square) and 47.6% (Nagelkerke *R*-squared) of the variance in functional impairment. The model correctly classified 77.5% of the cases. As shown in Table 3, the variables contributing to the model were: HAM-D total score (Beta = 0.39; Wald 32.56; *p* < 0.01; OR = 1.48), lifetime psychotic symptoms (Beta = 1.07; Wald = 4.77; *p* = 0.03; OR = 2.91), working memory IQ (Beta = −0.04; Wald = 7.52; *p* < 0.01; OR = 0.95), Wisconsin Card Sorting Test (WCST) number of categories (Beta = −0.35; Wald = 4.17; *p* = 0.04; OR = 0.70) and CVLT short cued recall (Beta = −0.38; Wald = 5.52; *p* = 0.02; OR = 0.68). Table 3 summarizes all the significant variables in the model. 

## 4. Discussion

This report aimed at identifying factors associated with functional recovery in a sample of euthymic patients with BD. Among all the clinical variables introduced in the model, lifetime psychotic symptoms is the one with the highest odds ratio, followed by the total score in the HAM-D. The present results also highlight the importance of preserving cognition in order to ensure better functional outcomes, since patients with lower scores in some executive functions (working memory index and WCST number of categories) and in verbal memory (CVLT short cued recall) are less likely to achieve functional recovery.

Lifetime psychosis appears as the most determinant variable in the model. In the present study, patients with lifetime psychosis symptoms were less likely to achieve a good functional outcome when compared to patients who had not experienced these symptoms. Psychosis occurs frequently in BD, approximately between 60–90% of patients have a lifetime history of psychotic symptoms [23,24], which is in line with the prevalence found in the present sample (up to 67%). Psychotic symptoms vary between patients and several factors could play a role including genetic predisposition, brain structure, substance abuse and family history of BD, among others [25,26]. The presence of active psychosis seems an important cross-diagnostic factor in BD that not only impacts on functional outcome, but also can be associated with poor cognitive performance [27,28]. However, the role of lifetime psychotic symptoms is still a matter under discussion with some authors reporting a negative impact of psychotic history both on psychosocial functioning and on cognition [29,30,31,32,33]; while others could not report this association [34,35,36,37]. Also, some scientists suggest that the two groups (patients with lifetime psychosis vs. non-psychosis) could be neuropathologically distinct, with smaller medial temporal, cingulate and lateral prefrontal volumes seen in patients with lifetime psychosis symptoms compared to those without [38]. Probably, the best way to prevent psychotic symptoms in BD and to ensure a good psychosocial functioning is to provide an adequate prophylactic treatment that prevents relapses, especially manic episodes, which are more frequently associated with psychotic symptoms than depressive episodes. In this regard, lithium [39,40,41] together with psychoeducation [42,43] appear to be the most effective strategy to prevent both manic and depressive relapses.

Another clinical significant variable contributing to the model includes subthreshold depressive symptoms, which is probably the most consistently reported variable across literature as a key factor that influences functional outcome [44,45,46,47,48,49,50,51,52,53]. Even though patients were euthymic when evaluated, scoring 8 or below in the HAM-D, the presence of subthreshold symptoms represents a barrier to achieving good functional adjustment. This is in line with previous literature reporting that low levels of depressive symptoms can interfere with concentration, increase fatigue, diminish motivation, increase social withdrawal and weaken social relationships [45,49]. Moreover, subthreshold depressive symptoms, which are more common than subthreshold hypomanic symptoms, are also a major cause of relapse [54,55,56] and can increase the risk of suicide [57]. Hence, it is not surprising that this variable appears systematically related to functional outcome. The burden of subthreshold depressive symptoms goes beyond psychosocial functioning and can also interfere in quality of life, preventing patients from living their lives to the fullest [45,58]. Hence, approaches that tackle this kind of symptomatology are urgently needed. Patients with pervasive subthreshold depressive symptoms are a huge challenge in clinical practice. Adding further medication, like an antidepressant, might worsen the scenario, increasing the risk of causing a switch to mania [59,60]. To the best of our knowledge, only one atypical antipsychotic (quetiapine extended release 300mg) has shown to be effective at improving subthreshold depressive symptoms, however with no effects on psychosocial functioning [61]. Besides, few non-pharmacological studies have found to improve these subthreshold depressive symptoms. One pilot RCT study assessed the effect of a therapy (Eye Movement Desensitization and Reprocessing, EMDR) and it found that patients in the EMDR group improved both in depressive and hypomanic subthreshold symptoms [62]. Other results from a secondary study suggests that interventions aiming at improving psychosocial functioning, like functional remediation, could be a good option to reduce subthreshold depressive symptoms, at least in patients with bipolar II disorder [63]. Finally, other reports point out the beneficial effects of healthy life styles (nutrition, exercise and wellness) to improve the outcome of patients with BD, including the treatment of depressive symptoms [64,65]. Hence, the ingredients of the therapy to treat these persistent symptoms, along with the improvement of functional outcome, are still unknown. It is likely that involves a combination of several approaches following the model of marginal gains originally designed by the Team Sky [66] and adapted to BD by Nierenberg and colleagues [67]. This method consists of implementing a wide variety of small changes that have a substantial aggregated positive impact in the long-term [67]. In this regard, it is expected that little changes executed at different areas (pharmacotherapy, psychological interventions, etc.) could lead to the improvement of this symptomatology, and as a consequence, increase psychosocial functioning. Introducing multicomponent programs, which allow tackling different areas to improve at the same time, represent a promising tool to fight against subthreshold depressive symptoms. One example of these multicomponent programs is the integrative therapy, recently introduced by Reinares and colleagues [68], which, with the aim to cover different areas affected by the illness, combines therapeutic components of broader treatments in the same program, including psychoeducation for patients and family members, mindfulness training, promotion of healthy life style and cognitive/functional enhancement. This comprehensive and integrative approach may be a fair reflection of the principle above-mentioned: little changes in different areas can exert a significant positive impact in the long-term [67].

In relation to the neuropsychological performance, a total of three variables were found to significantly contribute to the logistic regression: two variables related with executive functions (working memory IQ and WCST number of categories) and another one related with verbal memory (CVLT short cued recall). In BD, neurocognitive impairment can appear practically in all cognitive domains and in a large proportion of patients with effect sizes ranging from small to medium effects [69]; moreover, many studies have linked neurocognitive impairment with functional outcome in euthymic patients with BD [7,44,70,71]; nonetheless, there is no consistency across studies as to which neurocognitive variables could best explain functional outcome. The most reported neurocognitive areas in literature include verbal learning and memory [7,44,70,72,73,74,75], executive functions [5,52,70,76,77,78,79,80,81] and attention [73,75,82,83]. More recently, the role of premorbid IQ has also been highlighted as a key neurocognitive variable playing a role in psychosocial functioning [7,51,84,85,86,87]. In some of these studies premorbid IQ is associated to the concept of cognitive reserve (CR). It is hypothesized that higher CR could protect against functional decline [87,88,89]. In fact, the introduction of the CR concept raises the possibility of developing novel therapies based on intellectually stimulating activities directed to provide resilience against disease progression, and more specifically to prevent cognitive decline if they are implemented in early stages of the disease [90,91]. Ultimately, the enhancement of cognition (or by guaranteeing its preservation) could also prevent from functional decline [90]. 

The lack of consistency across different studies when reporting which neurocognitive variables influence functional outcome could be in part attributed to the tools used to evaluate both neurocognitive performance and functional outcome. In this regard, the standardization of the tools to assess these different features of BD is urgently needed. In 2010, Yatham and colleagues already proposed a preliminary battery of cognitive tests to be used in research. Among the recommended tests, they included the CVLT to assess verbal learning and memory, or the use of the SCWT and WCST to assess executive functions [92]. More recently, the Targeting Cognition Task Force from the International Society for Bipolar Disorders (ISBD) leaded by Dr. Miskowiak (2017) [93] encouraged using the tests already proposed in 2010 by the ISBD-BANC (Battery for Assessment of Neurocognition) [92] and also added some recommendations as to measure psychosocial functioning. In this regard, the FAST, the Brief UCSD Performance-based Skills Assessment (UPSA-B) and the Virtual Reality Functional Capacity Assessment Tool (VRFCAT) are proposed as the best tools to track changes in this area. Even though these recommendations were done in the context of cognition trials, they could also be applicable to clinical research in BD. If all researchers followed the above-mentioned guidelines, it is likely that more consistent results could be reported in this field. 

Of note is that in the present study no demographic variable has been linked with functional recovery. Gender and age were significantly different between the two groups in the univariate analysis and in line with previous literature male gender and older age were associated with poorer functional outcome [7]; however, when entered in the logistic regression model these variables were no longer significant. Our data suggest that the clinical course, specifically lifetime psychotic symptoms and subthreshold depressive symptoms, along with the preservation of the cognitive skills are more important to avoid functional decline than other demographic non-modifiable variables that include, for instance, gender or age. 

The results derived from the present study should be interpreted with caution in light of several limitations. First, the cross-sectional nature of the study does not allow establishing causal relationships between the independent and dependent variables. In contrast, and to the best of our knowledge, this is one of the largest studies assessing a broad range of variables that could influence functional outcome in a sample of euthymic patients with BD and the present results might be taken into account to design future studies and interventions. Another limitation is that pharmacotherapy was not controlled for; hence, treatment effects on cognition and functioning have not been studied. Third, the evaluators who performed the functional assessment were blind to the neuropsychological outcome for the vast majority of patients comprising this sample; unfortunately, we cannot ensure that this condition was accomplished for all the participants assessed in this study. Finally, it is worth mentioning that the FAST scale is not a performance-based tool, like the UPSA-B. Instead, the FAST could be classified as a semi-objective scale since it is interviewer-administered and the scores are partially based on patients’ reports but also takes into account relevant information about psychosocial functioning reported by relatives, the clinician and/or clinical reports [91].

## 5. Conclusions

Despite these limitations, we might conclude that long-term therapeutic interventions to enhance psychosocial functioning should focus on two targets: the clinical course and the preservation of cognition. As regards to the clinical course, avoiding psychotic symptoms appears to be the most important factor to prevent functional decline. Given that these symptoms occur during mood episodes, it might be suggested that the prevention of relapses is crucial to guarantee good functional outcomes. Lithium, together with specific adjunctive psychological treatments such as psychoeducation appear to be the most effective strategy to prevent relapses and ensure good functional outcomes [26,41,42]. Another critical clinical variable is the presence of subthreshold depressive symptoms, its management still represents a challenge in clinical practice; it is likely that they might be addressed through multicomponent programs that allow tackling different areas, following the principle of implementing small accumulative changes [67]. Finally, the preservation of cognition might be achieved by enhancing CR from the very beginning of the illness, and specially focusing on executive functions and verbal memory, as they seem to play a critical role in psychosocial functioning. Future longitudinal studies are needed in order to confirm the present results. Moreover, including the assessment of genetics and neuroimaging of these two groups (functionally remitted vs. impaired) might help us to better understand the biological underpinnings that could occur under these two phenotypically distinct groups. 

## Figures and Tables

**Table 1:** Demographic and clinical characteristics of the sample. IQ—intelligence quotient; HAM-D—Hamilton Depression Rating Scale-17; FAST—Functioning Assessment Short Test.

	Functionally Remitted (*n* = 221) Mean (SD)	Functionally Impaired (*n* = 199) Mean (SD)	*t* Student (*p* Value)
Age	38.4 (11.1)	44.5 (10.1)	−5.85 (<0.001)
Years of education	14.3 (3.3)	14.1 (3.9)	1.38 (0.16)
Estimated IQ	108.4 (8.8)	108.6 (9.7)	−0.16 (0.52)
YOUNG total score	1.2 (1.7)	1.4 (1.8)	−1.29 (0.19)
HAM-D total score	2.1 (2.2)	5.1 (2.9)	−11.11 (<0.001)
FAST total score	10.5 (6.6)	32.6 (8.9)	−28.5 (<0.001)
Chronicity (years of illness)	14.2 (10.3)	17.5 (11.1)	−3.1 (0.02)
Number of manic episodes	2.1 (2.6)	2.4 (3.1)	−1.10 (0.27)
Number of depressive episodes	4.6 (8.3)	6.7 (10.8)	−2.17 (0.03)
Number of hospitalizations	1.6 (1.7)	2.1 (2.4)	−2.36 (0.02)
	*n* (%)	*n* (%)	Chi (*p*)
**Gender (women)**	116 (52.5)	80 (40.6)	5.9 (0.01)
Diagnosis (type I)	173 (79.7)	142 (72.1)	3.31 (0.08)
Lifetime rapid cycling	15 (9)	26 (15.9)	3.5 (0.07)
Lifetime psychotic symptoms	142(66)	131 (67.2)	0.06 (0.83)
Family affective psychiatric history	109 (66.9)	118 (71.5)	0.83 (0.40)

**Table 2:** Neuropsychological performance in the two patient groups. TMT-A—Trail Making Test-part A; TMT-B—Trail Making Test-part B; CVLT—California Verbal Learning Test; SCWT—Stroop Color-Word Interference Test; WSCT—Wisconsin Card Sorting Test.

	Functionally Remitted (*n* = 221) Mean (SD)	Functionally Impaired (*n* = 199) Mean (SD)	*t* Student (*p*)
IQ Processing Speed	104.3 (17.3)	100.7 (14.3)	2.2 (0.02)
IQ Working Memory	100.5 (12.9)	95.6 (14.9)	3.5 (<0.01)
WCST categories	5.4 (1.4)	4.5 (1.9)	5.1 (<0.01)
WCST perseverative errors	11.8 (10.6)	18.1 (14.9)	−4.8 (<0.01)
TMT-A	27.8 (9.5)	37.6 (18.1)	−6.8 (<0.01)
TMT-B	70.7 (37.6)	110.6 (75.8)	−6.7 (<0.01)
CVLT #total words	56.5 (10.8)	50.9 (13.7)	4.6 (<0.01)
CVLT short-free recall	12.1 (2.9)	11.1 (6.1)	2.2 (0.02)
CVLT short-cued recall	13.0 (2.5)	11.8 (3.2)	4.2 (<0.01)
CVLT delay free recall	12.6 (2.8)	11.8 (7.3)	1.4 (0.15)
CVLT delay cued recall	13.2 (2.4)	12.1 (3.1)	4.1 (<0.01)
SCWT interference	52.6 (6,4)	52.3 (7.6)	0.47 (0.63)
Osterrieth Rey Figure	19.2 (4.8)	17.3 (5.3)	3.7 (<0.01)
Phonemic fluency	36.0 (9.7)	33.7 (10.2)	2.3 (0.02)
Animal naming	20.2 (4.5)	18.3 (5.6)	3.9 (<0.01)

**Table 3:** Logistic regression to identify the variables that best predict functional recovery.

Variables	Wald	*p* Value	OR	95% CI
Hamilton Depression total score	32.56	<0.01	1.48	1.29–1.70
Lifetime psychotic symptoms	4.77	0.03	2.91	1.11–7.54
Working Memory IQ	7.57	<0.01	0.95	0.93–0.98
WCST number of categories	4.17	0.04	0.7	0.50–0.98
CVLT short-cued recall	5.52	0.02	0.68	0.49–0.93

WCST: Wisconsin Card Sorting Test; CVLT: California Verbal Learning Test; OR: Odds ratio; CI: Confidence Interval.
